# False-positive rates in two-point parametric linkage analysis

**DOI:** 10.1186/1753-6561-8-S1-S110

**Published:** 2014-06-17

**Authors:** Silke Szymczak, Claire L Simpson, Cheryl D Cropp, Joan E Bailey-Wilson

**Affiliations:** 1 Statistical Genetics Section, Inherited Disease Research Branch, National Human Genome Research Institute, National Institutes of Health, 333 Cassell Drive, Suite 1200, Baltimore, MD 21224, USA; 2 Current address: Institute of Clinical Molecular Biology, Christian-Albrechts-University Kiel, Am Botanischen Garten 11, 24118 Kiel, Germany

## Abstract

Two-point linkage analyses of whole genome sequence data are a promising approach to identify rare variants that segregate with complex diseases in large pedigrees because, in theory, the causal variants have been genotyped. We used whole genome sequence data and simulated traits provided by Genetic Analysis Workshop 18 to evaluate the proportion of false-positive findings in a binary trait using classic two-point parametric linkage analysis. False-positive genome-wide significant log of odds (LOD) scores were identified in more than 80% of 50 replicates for a binary phenotype generated by dichotomizing a quantitative trait that was simulated with a polygenic component (that was not based on any of the provided whole genome sequence genotypes). In contrast, when the trait was truly nongenetic (created by randomly assigning affected-unaffected status), the number of false-positive results was well controlled. These results suggest that when using two-point linkage analyses on whole genome sequence data, one should carefully examine regions yielding significant two-point LOD scores with multipoint analysis and that a more stringent significance threshold may be needed.

## Background

The development of low-cost, high-throughput sequencing technologies has reignited the interest in family-based study designs for finding causal genes in rare and common diseases. Linkage analysis was designed for the investigation of rare variants of large effect and is robust to allelic heterogeneity. Most researchers are familiar with using multipoint methods for assessing linkage, which leverage cosegregation of a marker-haplotype with the trait in a family as a proxy for the location of causal variants. Whole exome sequencing (WES) and whole genome sequencing (WGS) of several or all individuals in a pedigree has now become feasible. These data provide information about genetic variability at a very high resolution. Multipoint linkage analyses of such dense sets of markers are time-consuming, do not scale well to millions of markers, and intermarker linkage disequilibrium (LD) can cause inflated type I error rates when pedigree founders are not genotyped [1]. A powerful and time-efficient method to detect dominant or codominant causal variants of large effect is two-point (sometimes called single-point) linkage analysis [2]. This approach analyzes each variant separately, reduces the computational load and enables parallel processing on a large scale. If the causal variant in a family has been observed reliably in the WES or WGS data, then that variant should yield the maximum two-point log of odds (LOD) score at a recombination fraction (θ) of zero across the genome in the family being studied. Other nearby variants should also yield high two-point LOD scores at θ near zero [1,3]. However, it is an open question whether the standard significance threshold of 3.3 for a genome-wide significant (GWS) LOD score [4] (which was based on the number of independent tests using multipoint linkage for an infinitely dense map) will control two-point false-positive rates to 1 in every 20 genome scans when several million variants are analyzed. Consequently, we investigated the proportion of false-positives applying classic two-point parametric Elston-Stewart linkage analysis using WGS data in extended pedigrees provided by Genetic Analysis Workshop 18 (GAW18) [5]. We used two phenotypes simulated under the null hypothesis of no linkage to any of the genetic variants in the data: (a) a binary trait generated by dichotomizing a quantitative trait simulated with a polygenic component but no linkage to any markers, and (b) a binary phenotype simulated under a complete null hypothesis of no linkage and no genetic effect on the trait.

## Methods

### Data

Genotype data for all 8,348,674 sequenced and imputed single nucleotide variants (SNVs) on the odd-numbered chromosomes were analyzed. There were 959 individuals with genotypes; 430 individuals without genotype data were added to provide complete pedigree information for linkage analysis, and one member was removed from each of two monozygotic twin pairs. There were 413 founders: 108 had genotypes and phenotypes, 9 had genotypes but no phenotypes, and 296 had neither. Because our goal was to estimate the false-positive rate under the null hypothesis of no linkage, we used two phenotypes that were simulated independently of any genotyped SNVs in the GAW18 data set. We requested the details of the simulation, and therefore knew the generating model of the simulated traits. The first analyzed phenotype was a binary trait based on the provided quantitative phenotype Q1 that was simulated by the GAW18 data providers to allow estimation of false-positives. Q1 was simulated as a normally distributed quantitative trait correlated among family members with heritability = 0.68, but was not influenced by any of the genotyped SNVs [5]. Mean levels of Q1 were simulated to decrease with age. To create our binary trait, we assumed that disease risk increased with age, and that affecteds would have low Q1 values. Therefore, our trait was dichotomized by estimating the 20% quantile of the distribution in all founders from all 200 replicates. All individuals with a Q1 value smaller than the estimated quantile were classified as affected and as unaffected otherwise. We simulated the second binary phenotype under a complete null hypothesis by randomly assigning affected-unaffected status to members of the families based on a disease prevalence of 10%. For both phenotypes, 50 replicates of the phenotype were analyzed.

Several pedigrees contained loops that cause problems for likelihood estimation in the linkage analysis program we used. Because our goal was to estimate false-positive rates under the null (and not to determine power to detect linkage or to detect an actual causal gene), we chose very simple strategies for breaking these loops to produce simple pedigrees. Different approaches to break loops were applied depending on their complexity. Removing the unaffected individual T2DG2501049 in pedigree 25 broke a loop caused by a woman with children from two brothers. The simple loops in pedigrees 2 and 3 were removed by duplicating an unaffected individual (T2DG0200031 and T2DG0300138) with both parents and children in the pedigree. Duplicated individuals had the same genotype and phenotype data but modified family relationships. Pedigrees 6 and 7 were excluded because their more complicated loops could not have been broken with the simple approaches used for the other pedigrees. Thus we had a set of simple pedigrees that allowed analysis of false-positive rates of two-point parametric linkage. In analyses of real data, use of a program that can analyze large pedigrees with loops intact would have optimal power.

### Two-point parametric linkage analysis

Allele frequencies of all SNVs were estimated with PLINK [6], using founders only, which is more accurate for common than rare variants given the many missing founders. For SNVs with no minor allele in the founders, minor allele frequency (MAF) was set to 0.0001. Two-point parametric Elston-Stewart linkage analysis was performed using the R package paramlink version 0.6-1 (http://cran.r-project.org/web/packages/paramlink/index.html). A dominant model was chosen because no, or only a very low number of, rare allele homozygotes are observed for rare SNVs and because we were seeking to evaluate type I error rate rather than power. In a real analysis, one could either use a model from segregation analysis or could utilize dominant, recessive, and additive models with reduced penetrance [7] and adjust the significance threshold for the additional analyses [8-13]. Penetrances were specified as 0.05 and 0.5 for genotypes dd and DD/Dd, respectively [7], with a frequency of 0.01 for allele D. The classic LOD score threshold of 3.3 was used for GWS linkage to evaluate the results [4]. The GWS LOD score means that under the null hypothesis only 1 of 20 replicates would have at least one marker with a LOD score equal to or larger than 3.3 [3].

## Results

Results are presented separately for the two different unlinked phenotypes.

### Binary trait based on Q1

**Figure 1:**
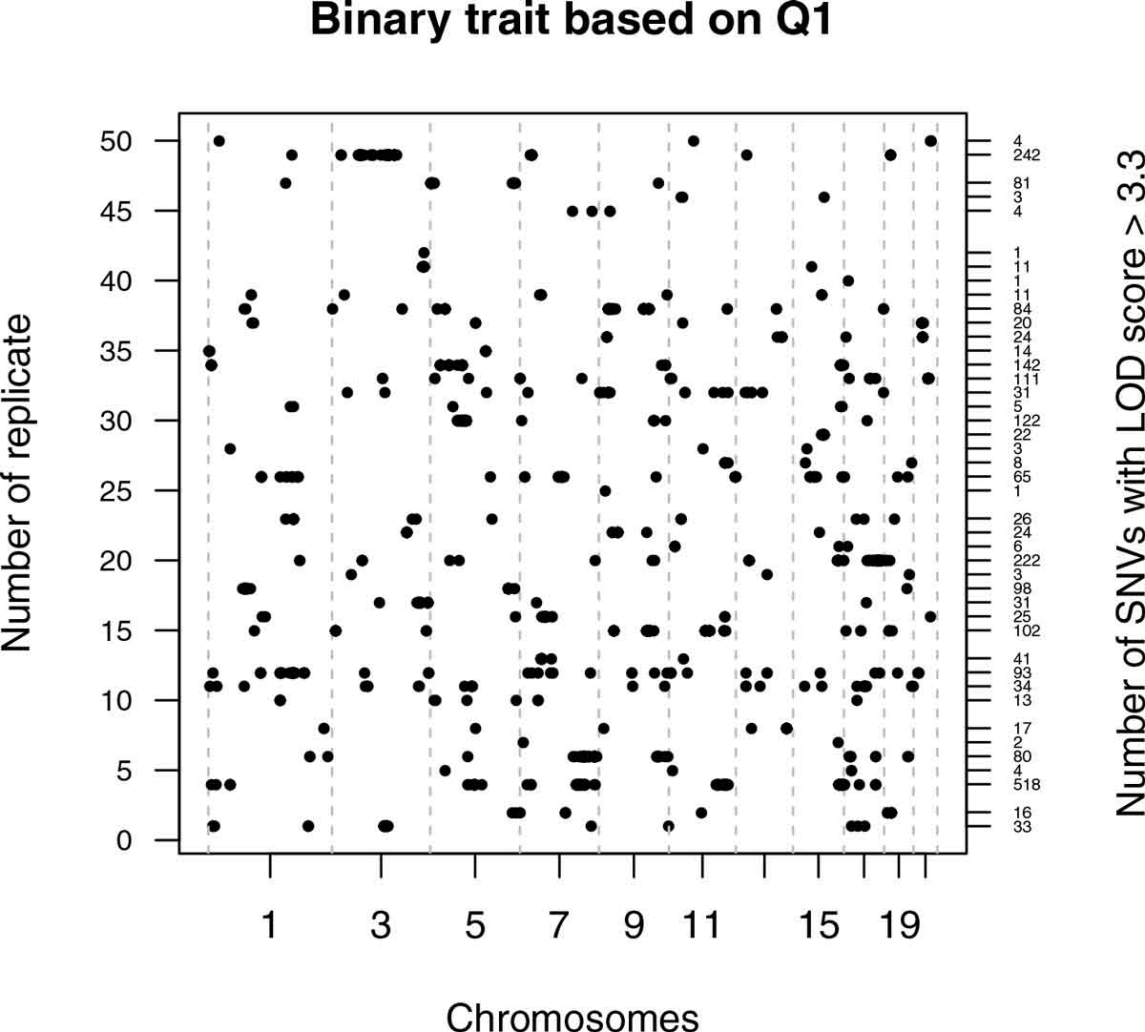
**Genome-wide significant LOD scores in each replicate for the binary trait based on Q1**. Each point represents the location of a GWS LOD score (x-axis) in a specific replicate. (y-axis). Each replicate is identified by its replicate number on the left axis and numbers of significant SNVs in each replicate are given on the right.

For the binary trait based on the provided quantitative null trait Q1, 43 of 50 replicates showed at least one GWS LOD score, with values ranging from 3.3001 to 5.685 (Figure 1). On average 55 (range: 1 to 518) false-positive significant SNVs were found per replicate, distributed over all the provided chromosomes. Each of the total 2398 significant SNVs was significant in only one replicate. Of these SNVs, 138 had an estimated MAF < 0.05, with 28 having a MAF < 0.01, and only 12 had MAF set to 0.0001. Of the significant SNVs, 1008 have one or two families with a LOD score greater than 1.9, the classic threshold for suggestive linkage [4], whereas only 70 of the significant SNVs have no families with LOD scores > 1 and have between two and five families with LOD scores > 0.5.

### Binary trait under the complete null hypothesis

In contrast, for the binary trait simulated under a complete null hypothesis (random assignment of affected/unaffected status), only two out of the 50 replicates had GWS results. In one replicate, one SNV on chromosome 1 had a combined LOD score of 3.336 that was driven by a LOD score of 3.334 in family 3. The MAF was estimated as 0.0085, and all seven affecteds in family 3 were heterozygous for the rare allele. Only three of 53 unaffecteds in this family were heterozygotes. In another replicate, 19 SNVs in a 4.6 Mb region on chromosome 3 had combined LOD scores between 3.308 and 3.751, with family 5 having the largest family-specific LOD scores, ranging from 2.128 to 2.578. These SNVs were in two blocks with nearly complete LD, and estimated MAF was 0.389 on average. Again, all affecteds, or all but one affected, were heterozygous or homozygous for the rare allele in family 5.

## Discussion

Linkage analysis fell out of favor in the genome-wide association studies era, but the power of families can be well leveraged in sequencing studies. The advent of large-scale WES and WGS studies has rekindled interest in family-based analysis designs [1,14]. Family-based designs offer protection from population stratification, and Mendelian consistency checking provides additional validation of observed rare or novel variants. Although a variant may be rare in the population overall, large pedigrees ascertained for a particular trait of interest will be enriched for rare variants with some effect on the trait [14], and there are likely to be several individuals in a family with a rare causal variant. Thus the number of individuals required for sufficient power to detect rare variants of moderate to large effect is considerably lower in family studies as opposed to studies of unrelated individuals, especially when there is also substantial locus heterogeneity [2,14-16]. However, it was unclear whether the significance thresholds [4], which were adequate for multipoint analysis or two-point linkage of hundreds to thousands of marker loci, would be appropriate for WGS data with millions of SNVs.

In this study we were interested in type 1 error rates of two-point linkage analysis of a binary trait under the null hypothesis of no linkage. GAW18 provided only one quantitative trait simulated under the null hypothesis of no linkage (simulated to have true genetic causal variants, but not on the provided chromosomes) so we chose to dichotomize this trait; we also simulated our own qualitative trait that had no genetic effects whatsoever. As our purpose was evaluation of false-positive rates for traits under the null, issues of power do not apply, and this approach is reasonable. However, we do not recommend dichotomization of a quantitative trait in real data because linkage analysis is generally more powerful for a quantitative trait compared to a binary dichotomization of that trait. Incorporating covariates into the linkage analysis can also improve power in a real study especially for established risk factors.

Our analysis revealed very different results for the two null phenotypes. We observed a large number of false-positive results for the polygenic trait, although it was simulated without using any of the provided genotypes and was unlinked to the provided chromosomes. Most of the false-positive results observed for this trait involved SNVs with relatively common minor alleles, and only 12 of the 2398 significant SNVs across all 50 replicates had arbitrary MAF of 0.0001. Although incorrect specification of both the trait model and the marker allele frequencies can inflate type I error rates, estimation of marker allele frequencies from the data (as we did here) has been shown to control false-positive rates even when the trait model is misspecified, so model misspecification is not likely the cause of the type 1 error rate inflation observed here. Differences in pedigree structure and model misspecification also appeared to have little effect on false-positive rates of two-point LOD score linkage of complex traits in a study of a single marker with 8 equifrequent alleles [17]. The simulated polygenic component introduces genetic correlation between family members based on their degree of relationship so that the trait is not segregating randomly. Because each family for this simulated trait is segregating more than eight million SNVs under the same Mendelian constraints that cause the simulated trait correlations, the inflated false-positive rate for this trait is most likely attributable to chance cooccurrence of the same correlations between relative pairs for the trait and a variant [16]. Thus the problem here is most likely the extremely large number of tests being performed in each genome scan. It is possible that some inflation is a result of inaccurate estimates of SNV allele frequencies, so in real analyses, one should reestimate these frequencies using maximum likelihood estimation for any significantly linked variants if some founders are ungenotyped.

In contrast, the number of false-positive results based on the classic LOD score threshold of 3.3 is well controlled if affected-unaffected status is randomly assigned (ie, no genetic component exists), even when more than eight million SNVs are analyzed by two-point parametric linkage analysis.

Both two-point and multipoint linkage analysis can detect linkage to a region containing a causal variant even if the causal variant was not genotyped. Even though we only evaluated LOD scores at θ = 0, in a real analysis, one should also obtain the LOD score at the maximum likelihood estimate of θ to detect SNVs very close to a causal variant. One must be careful interpreting a two-point analysis because the most significant two-point LOD may not indicate the actual causal variant, but simply the closest variant to the untyped causal variant as, for example, when the causal variant is not analyzed because it is in a region with low coverage or was filtered out because of bad quality. One should examine the strongest linkage region for variants dropped because of low quality and possibly resequence the most strongly linked region.

## Conclusions

This study of false-positive rates in WGS data simulated under the null hypothesis of no linkage to any chromosomes provided in the data set suggests that a higher LOD score threshold may be required when analyzing a complex trait with nonzero heritability. However, more extensive simulations are required before such new thresholds can be proposed. Reestimating allele frequencies and performing multipoint linkage in regions showing significant two-point scores may help to resolve this problem. Single significant SNVs with no supporting linkage to nearby variants may be eliminated by the multipoint analysis. This remains an area of future research.

## Competing interests

The authors declare that they have no competing interests.

## Authors' contributions

CLS, SS, and JEBW designed the study; CLS, SS, and CDC conducted analyses. CLS and SS drafted the manuscript. All authors read and approved the manuscript.
